# On the Physiology of Normal Swallowing as Revealed by Magnetic Resonance Imaging in Real Time

**DOI:** 10.1155/2014/493174

**Published:** 2014-02-12

**Authors:** Arno Olthoff, Shuo Zhang, Renate Schweizer, Jens Frahm

**Affiliations:** ^1^Department of Otorhinolaryngology, Phoniatrics and Pedaudiology, Universitätsmedizin, Georg-August-Universität Göttingen, Robert-Koch-Straße 40, 37075 Göttingen, Germany; ^2^Biomedizinische NMR Forschungs GmbH am Max-Planck-Institut für Biophysikalische Chemie, 37077 Göttingen, Germany

## Abstract

The aim of this study was to assess the physiology of normal swallowing using recent advances in real-time magnetic resonance imaging (MRI). Therefore ten young healthy subjects underwent real-time MRI and flexible endoscopic evaluations of swallowing (FEES) with thickened pineapple juice as oral contrast bolus. MRI movies were recorded in sagittal, coronal, and axial orientations during successive swallows at about 25 frames per second. Intermeasurement variation was analyzed and comparisons between real-time MRI and FEES were performed. Twelve distinct swallowing events could be quantified by real-time MRI (start time, end time, and duration). These included five valve functions: oro-velar opening, velo-pharyngeal closure, glottal closure, epiglottic retroflexion, and esophageal opening; three bolus transports: oro-velar transit, pharyngeal delay, pharyngeal transit; and four additional events: laryngeal ascent, laryngeal descent, vallecular, and piriform sinus filling and pharyngeal constriction. Repetitive measurements confirmed the general reliability of the MRI method with only two significant differences for the start times of the velo-pharyngeal closure (*t*(8) = −2.4, *P* ≤ 0.046) and laryngeal ascent (*t*(8) = −2.6, *P* ≤ 0.031). The duration of the velo-pharyngeal closure was significantly longer in real-time MRI compared to FEES (*t*(8) = −3.3, *P* ≤ 0.011). Real-time MRI emerges as a simple, robust, and reliable tool for obtaining comprehensive functional and anatomical information about the swallowing process.

## 1. Introduction

Oropharyngeal dysphagia is a frequent sequelae caused by neuromuscular and neurological diseases or by structural and organic lesions of the oropharyngeal tract as well as in elderly patients [1, 2]. The resulting deglutitive malfunctions include wrong bolus direction with penetration and aspiration, insufficient bolus clearance with retentions in the vallecula and piriform sinuses, and prolonged or disturbed timings of the swallowing events during deglutition [3]. Because the concise temporal succession of the various swallowing phases is critical for the direction and clearance of the bolus, it would be advantageous to have a diagnostic tool to detect and characterize them with good contrast and sufficient resolution.

At present videofluoroscopy reflects the gold standard in the diagnosis of deglutitive malfunctions. It offers dynamic images in the sagittal plane illustrating the complete course of deglutition but implies X-ray radiation exposure to patients. A coronal plane is usually also needed to exclude laryngeal penetration, whereas a view in the axial plane is not possible. Timing evaluation of the physiological events during swallowing relies exclusively on bony anatomic landmarks due to the limited visualization of soft tissues. On the other hand, flexible endoscopic evaluations of swallowing (FEES) offer an axial view and allow for pre- and postdeglutitive analyses that are valuable to clinical diagnosis and therapeutic planning in swallowing disorders. However, the “white out” at the moment of swallowing makes it difficult to reveal silent intradeglutitive aspiration and thus excludes the possibility for timing analyses. Recently, high-resolution manometry was reported to yield a robust predictor of aspiration in the diagnosis of oropharyngeal dysphagia [4].

Preliminary studies using magnetic resonance imaging (MRI) demonstrated potential for the evaluation of swallowing events and maneuvers in a supine position [5–9]. However, such trials usually suffered from limited spatial and temporal resolution. An axial plane was normally not considered and the analysis of the event timings was hardly possible due to relatively poor image quality. Fortunately, recent advances in the field of real-time MRI not only achieved high image quality, excellent tissue contrast, and virtually no motion artifacts, but also a spatiotemporal resolution comparable to that of conventional videofluoroscopy [10, 11]. A first study focusing on the technical aspects successfully visualized the dynamics of the oropharyngeal structures during normal swallowing and revealed great potential for providing noninvasive access to the process of deglutition [12]. Here, we attempt to quantitatively assess the temporal events that define the individual physiological steps of swallowing in normal subjects by real-time MRI. The results are compared to FEES of the same subjects as well as to literature findings using videofluoroscopy.

## 2. Materials and Methods

### 2.1. Subjects

Ten healthy volunteers (4 men, 6 women) with a mean age of 28 ± 3 years (SD = standard deviation) and a range from 26 to 35 years were recruited from the local university. The selection criteria involved no history or presence of dyspnea, dysphonia, and dysphagia. They were fulfilled based on personal medical history and FEES examination of all subjects by an experienced otorhinolaryngologist (A. O.). The Institutional Review Board approved the study and all participants gave written informed consent prior to examination. MRI data of the same subjects have been used in a previous publication introducing the real-time technique [12].

### 2.2. FEES

Transnasal FEES was performed in a sitting position with a typical temporal resolution of 25 frames per second (fps). An oral bolus of one teaspoon (5 mL) green-colored pear pie was used to ensure a clear contrast to the tissues of the oropharyngeal tract. The flexible endoscope (Olympus ENF, Hamburg, Germany) was connected to a camera (Olympus visera OTVS-7, Hamburg, Germany) and the recorded videos were stored in a hard disk (rpSzene, Rehder & Partner GmbH, Hamburg, Germany) for further evaluation.

### 2.3. Real-Time MRI

Dynamic MRI of deglutition in real time was performed with the use of a 3 Tesla MRI system (Tim Trio, Siemens Healthcare, Erlangen, Germany). The recently introduced real-time MRI technique [12] is based on a highly undersampled radial fast low-angle shot (FLASH) acquisition [13] in combination with image reconstruction by regularized nonlinear inversion [14]. Online reconstruction at about 17 fps was accomplished by running a parallelized version of the algorithm on a computer equipped with 8 graphical processing units that bypassed the conventional image reconstruction pipeline of the commercial MRI system [15, 16].

Subjects were examined in a supine position with a combination of a small flexible coil (Siemens Healthcare, Erlangen, Germany) covering the lower face and a bilateral 2 × 4 array coil (NORAS MRI products, Hoechberg, Germany) centered to the thyroid prominence on both sides of the neck. Successive T1-weighted images (repetition time TR = 2.17 ms, echo time TE = 1.44 ms, flip angle 5°, field of view 192 × 192 mm^2^) were acquired with an in-plane resolution of 1.5 × 1.5 mm^2^ and a slice thickness of 10 mm in a midsagittal, oblique coronal, and oblique axial orientation. The total image acquisition time was 41.23 ms, which yielded a true temporal resolution of 24.3 fps without data interpolation or combination.

Pineapple juice was used as oral contrast agent due to its content of paramagnetic manganese, which leads to a bright signal in T1-weighted images [17]. Prior to the examination the pineapple juice was thickened with starch (Quick & Dick, Pfrimmer Nutricia, Erlangen, Germany) to improve the visibility of tissue actions and movements. The thickened bolus (5 mL) was then given to the subject by an otorhinolaryngologist (A. O.) during the examination. After starting the dynamic image recording the subject was asked to swallow in a natural manner at a comfortable rate. Because of the complex movements of the relevant anatomical structures during deglutition, movies of individual swallows were recorded twice in the same sagittal plane and 4 to 5 times in a coronal as well as in multiple axial planes (5 mm shifts) to cover the entire region of interest. Further details of the imaging method and examination procedure were presented in a preceding article [12].

### 2.4. Swallowing Events

To evaluate the swallowing events and their quantitative timings, the viewing software OsiriX (open-source software: http://www.osirix-viewer.com/) [18] and iMovie HD (version 6.0.3, Apple Computer Inc., USA) were used for real-time MRI movies and FEES videos, respectively. Both programs allowed for a “frame by frame” evaluation of the data and served to characterize and quantitatively analyze distinct deglutition events, particularly their start and end points, by one otorhinolaryngologist (A. O.). For the analysis of valve functions the concept of the “six-valve model” proposed by Logemann from videofluoroscopic observations [3] was adopted as reference and compared to the present findings. To assess the visibility of each defined event, the timings of respective start and end points were classified in a dichotomic manner (yes or no). The results served to calculate a visibility ratio (in percentage) for each image orientation for all subjects. Only the orientation offering the highest visibility ratio was used for a determination of quantitative timings.

### 2.5. Quantitative Timings

Absolute durations of each event were calculated by subtracting the timings of their end and start point images. For relative timings a reference event had to be chosen. Although the opening of the esophagus has been employed in previous studies [12, 19], the ambiguous appearance of the sphincter during bolus transport in the MRI movies impaired a clear definition of the event. Here, the oro-velar opening or velum elevation from dorsum of the tongue could always be detected as a distinct landmark during deglutition and, therefore, served as temporal reference for all other events with its start time set to zero.

### 2.6. Statistics

Two-way repeated measures ANOVAs with the factors “measurements” and “events” were conducted to investigate possible differences for the start times, end times, and durations of defined events in two repetitive sagittal measurements. Post hoc paired *t*-tests between these measurements were conducted if applicable. For comparison between real-time MRI and FEES, the absolute timings of the same event were evaluated with a paired *t*-test.

## 3. Results

Real-time MRI movies were obtained from all subjects without any complications and complaints, while one FEES examination failed due to noncompliance of the subject to the transnasal procedure. Both MRI and FEES revealed normal deglutition and oromandibular function in all subjects. The use of a standardized real-time MRI examination protocol and a total in-room time of only about 15 minutes considerably reduced subject discomfort and facilitated the whole procedure.

### 3.1. Swallowing Events

**Figure 1:**
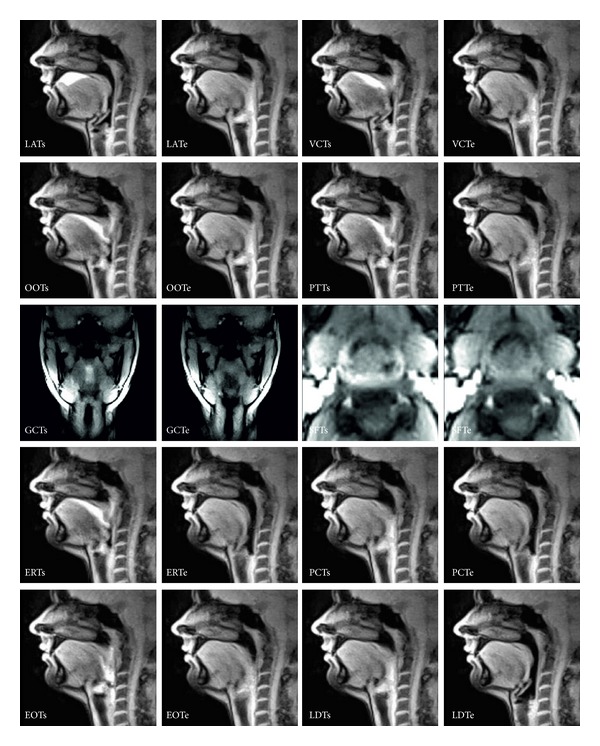
Physiological events of normal swallowing as seen by real-time MRI (27-year-old female). LAT: laryngeal ascent, VCT: velo-pharyngeal closure, OOT: oro-velar opening (start time defined as reference), PTT: pharyngeal transit, GCT: glottal closure, SFT: vallecular and piriform sinus filling, ERT: epiglottic retroflexion, PCT: pharyngeal constriction, EOT: esophageal opening, LDT: laryngeal descent (“s” and “e” refer to respective start and end times). The images are selected from respective movies (see Supplementary Movie 1 in the Supplementary Material available online at http://dx.doi.org/10.1155/2014/493174) at a resolution of 41.2 ms (24.3 frames per second) and sorted according to their temporal onset from top left to bottom right. For further details see text.

A total of 12 distinct swallowing events were detected by real-time MRI. They are characterized as 5 valve functions, 3 bolus transports, and 4 additional events. A detailed description is given in Table 1, while a typical example of all individual events is shown in Figure 1. Valve functions were defined according to the observation of their oral and pharyngolaryngeal apertures (i.e., valves) that play a functional role in deglutition. They represent the oro-velar opening (OOT), velo-pharyngeal closure (VCT), glottal closure (GCT), epiglottic retroflexion (ERT), and esophageal opening (EOT). Their start and end points were defined by the contact and separation of the corresponding valve tissues. For example, a wide opening of the soft palate from the dorsum of the tongue indicates the OOT start, while their subsequent contact marks the OOT end which could newly be identified in this study.

The three events that describe the passing of the bolus rather than the behavior of the valve refer to the oro-velar transit (OTT), pharyngeal delay (PDT), and pharyngeal transit (PTT). The OTT represents the bolus transport through the oro-velar valve, whose start and end were coincident with that of the OOT. The PTT was defined as the duration from the onset of the bolus head passing the oro-velar valve (start) to the point where the bolus tail passes the esophageal sphincter (end), while the PDT was the interval between the OOT start and the onset of the bolus head passing the oro-velar valve. Additional events included the laryngeal ascent (LAT), laryngeal descent (LDT), vallecular and piriform sinus filling (SFT), and pharyngeal constriction (PCT). The latter two events were again newly detected by real-time MRI.

### 3.2. Detectability

Table 2 summarizes the best orientations for the detection of individual events by real-time MRI. Excluding glottal closure and piriform sinus filling, the sagittal plane demonstrated the highest detectability yielding a visibility ratio of 95% for esophageal opening and 100% for all other events. Glottal closure was best seen in a coronal plane (87%), whereas the vallecular and piriform sinus filling were best detected in an axial plane (95%, as printed in bold type). The velo-pharyngeal closure, glottal closure, and pharyngeal constriction could also be detected in coronal or axial planes albeit with a slightly lower but nevertheless good visibility rate (>80%, as printed in bold italic type). These supplementary image orientations are expected to provide complementary information in subsequent clinical studies. In contrast to real-time MRI, FEES only visualized the velo-pharyngeal closure.

### 3.3. Quantitative Timings

**Figure 2:**
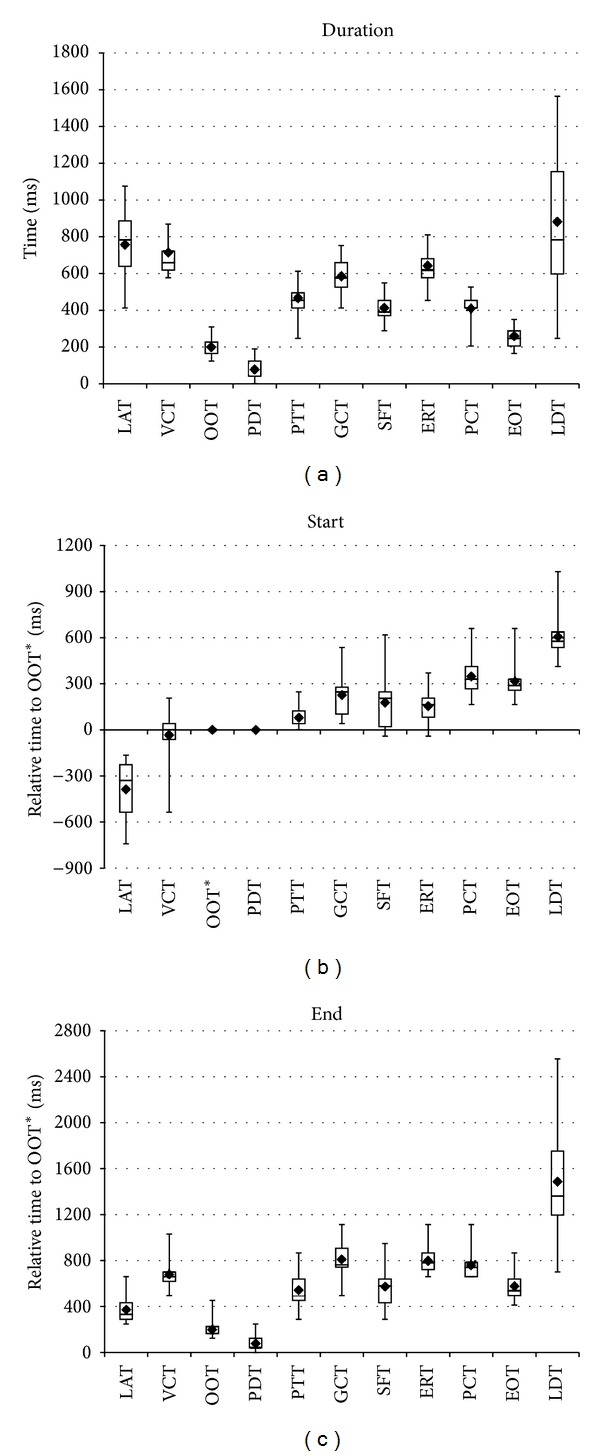
Durations, start times, and end times of distinct swallowing events as determined by real-time MRI (means and quartiles, 10 subjects). LAT: laryngeal ascent, VCT: velo-pharyngeal closure, OOT: oro-velar opening (start time defined as reference), OTT: oro-velar transit, PDT: pharyngeal delay, PTT: pharyngeal transit, GCT: glottal closure, SFT: vallecular and piriform sinus filling, ERT: epiglottic retroflexion, PCT: pharyngeal constriction, EOT: esophageal opening, and LDT: laryngeal descent.

Figure 2 summarizes the durations as well as start and end times (relative to OOT onset) of individual swallowing events as determined by real-time MRI. While the individual temporal accuracy is limited to the acquisition time of a single frame, that is, 41 ms, the mean durations averaged across subjects were OOT = 200 ± 83 ms (mean ± SD), VCT = 714 ± 147 ms, GCT = 586 ± 93 ms, ERT = 642 ± 130 ms, EOT = 261 ± 62 ms, OTT = 200 ± 83 ms (same as OOT), PDT = 78 ± 66 ms, PTT = 467 ± 117 ms, LAT = 757 ± 189 ms, LDT = 881 ± 410 ms, SFT = 413 ± 95 ms, and PCT = 410 ± 73 ms. Based on detectability, the relative timings of most start and end points were calculated from data measured in the sagittal plane. GCT timings were calculated via VCTs (detectable in both sagittal and coronal planes), while SFT timings were calculated via GCTs (detectable in both coronal and axial planes). This approach was validated by the strong correlation of the VTC durations for sagittal and coronal measurements (*r* = 0.8) and of the GCT durations for coronal and axial measurements (*r* = 0.63).

**Figure 3:**
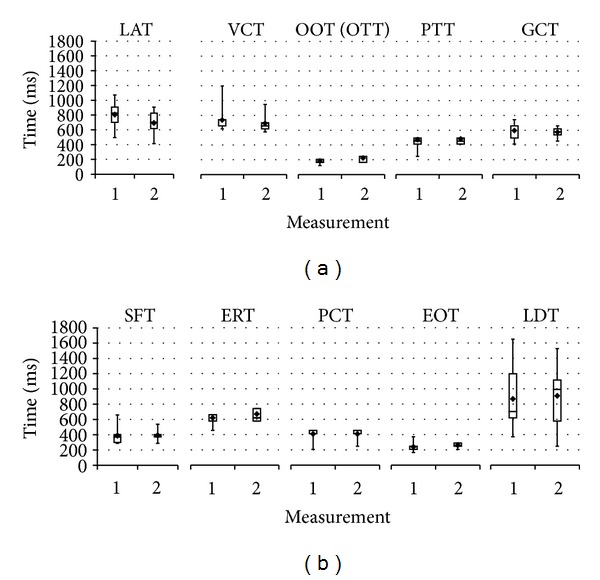
Durations of distinct swallowing events for two repetitive real-time MRI measurements (means and quartiles, 10 subjects). LAT: laryngeal ascent, VCT: velo-pharyngeal closure, OOT: oro-velar opening (start time defined as reference), OTT: oro-velar transit, PTT: pharyngeal transit, GCT: glottal closure, SFT: vallecular and piriform sinus filling, ERT: epiglottic retroflexion, PCT: pharyngeal constriction, EOT: esophageal opening, and LDT: laryngeal descent.

**Figure 4:**
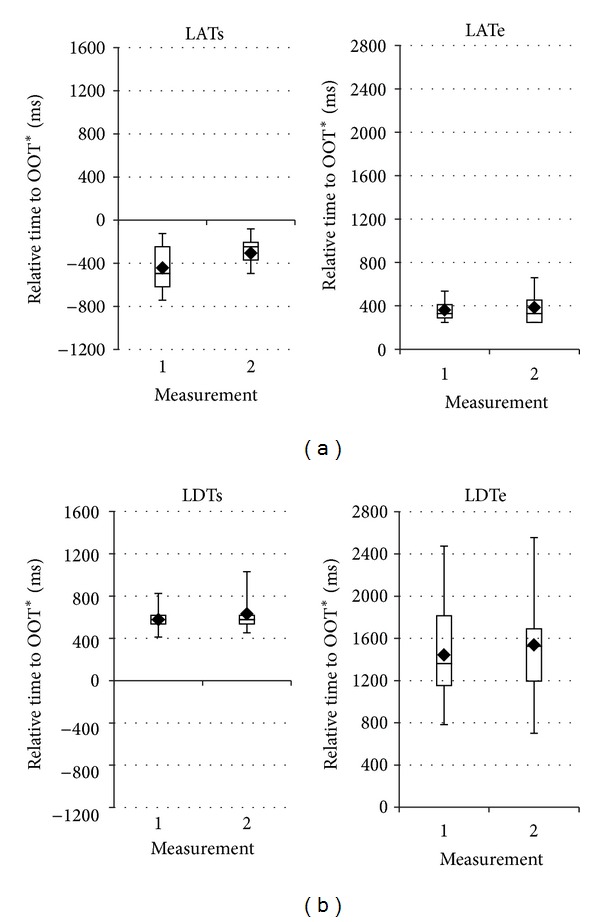
Start and end times of laryngeal ascent (LAT) and descent (LDT) for two repetitive real-time MRI measurements (means and quartiles, 10 subjects).

Data from the two repetitive sagittal measurements were compared with ANOVAs for durations (Figure 3) as well as relative start and end times. A marginally significant difference between two measurements can only be shown for the start time across the events (main effect “measurement”: *F*(1,8) = 5.0, *P* ≤ 0.055), but not for the duration or end time. In particular, this discrepancy in start time differs between events (“event × measurement”: *F*(7,56) = 2.9, *P* ≤ 0.011) and is significant only for LATs (*t*(8) = −2.6, *P* ≤ 0.031) and VCTs (*t*(8) = −2.4, *P* ≤ 0.046) (Figure 4).

### 3.4. Real-Time MRI versus FEES

The duration of the velo-pharyngeal closure was compared between real-time MRI and FEES, as it is the only event detectable by the latter. Sagittal and coronal real-time MRI studies resulted in 724 ± 144 ms and 690 ± 112 ms, respectively, which strongly correlate (*r* = 0.927) and do not differ from each other (*t*(8) = 1.8, *P* ≤ 0.117). However, these findings were significantly different from 591 ± 77 ms as detected by FEES for both sagittal (*t*(8) = −3.3, *P* ≤ 0.011) and coronal MRI planes (*t*(8) = −2.5, *P* ≤ 0.036).

## 4. Discussion

This study demonstrates that recent advances in real-time MRI [11, 12, 20, 21] offer a simple, robust, and well-tolerated access to the physiological details characterizing normal swallowing. The dynamic imaging approach allows for a comprehensive delineation of all deglutition events and a quantification of their temporal pattern at arbitrary image orientations. In fact, the used technique overcomes the long acquisition times of conventional MRI examinations and achieves a temporal resolution comparable to that of a typical videofluoroscopic or FEES measurement of deglutition. As a result, a more detailed depiction of tissue behavior and bolus transport has become possible.

When compared to Logemann's model [3], most valve function events detected by real-time MRI correspond well to previous findings by videofluoroscopy. A few differences exist for oro-velar opening, vallecula and piriform sinus filling, and pharyngeal constriction. In addition, because the bolus was passively given to the subject, the behavior of the lips and tongue—previously defined as valve 1 [3]—is not considered in the present study. The opening and closure of the oro-velar valve secure a safe bolus transport from oral to pharyngeal area and also indicate its efficiency. Therefore, the event of OOT was newly defined based on the present observations. Since vallecula and piriform sinus filling can only be seen in the axial plane, it was not observed in videofluoroscopy. As for pharyngeal constriction, Logemann stated that the pharyngeal wall is not observed as a “peristaltic wave” [19], but instead the movement of the tongue pushes the bolus forward. In contrast, we found two confluent peristaltic waves in all 10 subjects. The first one refers to passavant ridge that is caused by the contraction of the superior pharyngeal constrictor and occurs concurrently with velo-pharyngeal closure. The second wave refers to pharyngeal constriction and can be interpreted as a peristaltic lowering of the passavant ridge. It occurs almost concurrently with the opening of the cricopharyngeal sphincter and is followed by the esophageal peristaltic wave. This can be observed in both sagittal and coronal planes but is even better demonstrated in dynamic movie recordings (e.g., see Supplementary Movie 1). Such concurrent movements, namely, between VCT and passavant ridge as well as between EOT and PCT, work together with all temporally coordinated functions of individual valves to ensure a full clearance of the bolus in the aerodigestive tract and a solid protection of the airway.

For bolus transport events, the establishment of a landmark location appears to be a major challenge. So far, there is no generally accepted standard. Previous definitions refer to the “bolus head reaching the cross of the tongue base and posterior aspect of the mandible ramus” and “start of laryngeal elevation” to distinguish between oral transit, pharyngeal delay, and pharyngeal transit [3]. However, because the MRI signal arises from a cross-sectional slice rather than from overlapped structures in an X-ray projection image, the “cross of tongue base and mandible” cannot be seen in the movie recordings. At the same time, the tongue motion, inconsistently defined in previous studies [3, 11, 22, 23], greatly varies during oral bolus transport which renders it unreliable as landmark. Therefore, the oro-velar transit was newly defined as bolus passing through the oro-velar aperture, which is distinct and clearly visible. Its timing is identical to that of the oro-velar opening as the corresponding valve function.

Similar concerns apply to the laryngeal ascent, which was previously defined as a landmark for the pharyngeal transit and its timings (PTT). The present data show that the laryngeal ascent is one of only two events with a significant difference in the start time between two repeated measurements, indicating that various laryngeal movements are involved at the early phase of swallowing [12]. This can also be seen in the sagittal view of Supplementary Movie 1 and in Figure 2 revealing a large distribution of LAT start times and durations across subjects. Therefore, the well visible and distinct bolus head passing the oro-velar valve has been chosen as a consistent landmark describing the start of the pharyngeal transit.

Based on these definitions, a delay ranging from zero to 200 ms (78 ± 66 ms) was observed between the wide opening of the oro-velar valve (OOTs) with the bolus ready to enter the pharyngeal cavity and the onset of the bolus head passing the oro-velar valve (PTTs)—here defined as pharyngeal delay (PDT). Although using a different landmark, its timing is in accordance with earlier videofluoroscopy findings [23]. Whether this new “PDT” observed by real-time MRI provides a more accurate, reliable, and meaningful standard for studying the related mechanism and eventually resolving the literature discrepancy [3, 23, 24] remains to be seen in future clinical investigations.

Despite the limited number of subjects, our results clearly demonstrate that the sagittal plane is the most valuable orientation for the assessment of most physiological swallowing events. This also applies to conventional videofluoroscopy and may further simplify clinical applications by restricting the MRI examination to the sagittal plane at even shorter scan times. For example, this would particularly be favorable for patients suffering from dysphagia.

In the analysis of individual swallowing events, the choice of a landmark or reference is of great importance. So far, the beginning of the esophageal opening (EOTs) was taken as the reference [12, 19]. However, due to the low concentration and complex fluid dynamics of the bolus, a clear MRI identification of the moment when the complete bolus tail passes through the upper esophageal sphincter is rather difficult. In addition, MRI signals from saliva or tissues entering from neighboring areas (sections) during deglutition render this time point even more ambiguous. The same effect has contributed to a slightly lower detectability of EOT (95%) in real-time MRI. Therefore, the start time of the newly defined oro-velar transit (OTTs) which could readily be identified in all subjects was used as reference for the calculation of relative timings (Figure 1). While future clinical applications may include submental EMG recordings as an independent reference for a zero time point, such strategy was not considered in this pilot study.

Here the descriptive statistics has revealed different levels of variance associated with individual swallowing events across the subjects. In particular, LAT, VCT, and LDT show larger variation in durations whereas all the other events have no difference in relative timings and durations from one measurement to the other. This is better illustrated in Figure 3 for comparison of the durations between two repetitive real-time MRI sagittal measurements. Inspection of individual subject data shows that for VCT the difference is mainly caused by a single subject with an unusual earlier start time but no change in end time (not shown here). For LAT the significant difference stems from a variance in the start time (LATs), more specifically, a later start time in the second measurement in majority of the subjects, as seen in Figure 4. Such a difference is also observed for the end time of LDT but not its start time (LDTe in Figure 4) although it did not reach significance due to the large variance. The larger variance associated with LAT and LDT may be caused by variation of the repeated swallowing behaviors rather than by the measurement method. In fact, this may well indicate that LATs and LDTe belong to volitional elements while all rest events are reflexive, which implies again laryngeal movements may not be a proper and reliable landmark to study swallows timings.

**Figure 5:**
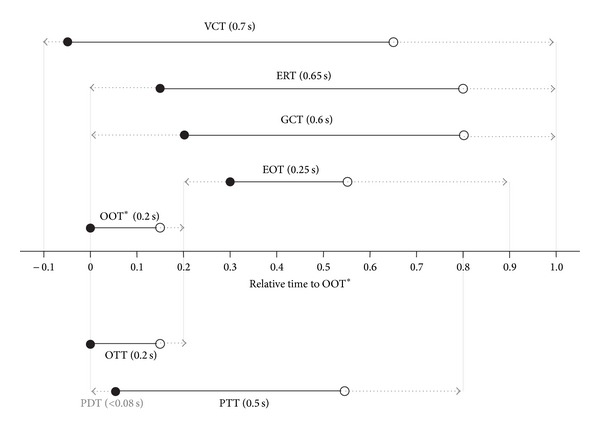
Temporal pattern of physiological events during normal swallowing. VCT: velo-pharyngeal closure, ERT: epiglottic retroflexion, GCT: glottal closure, EOT: esophageal opening, OOT: oro-velar opening (start time defined as reference), OTT: oro-velar transit, PDT: pharyngeal delay, and PTT: pharyngeal transit. Solid lines indicate durations with mean values in brackets (10 subjects), while circles represent respective start (solid) and end times (open). Arrows refer to maximal durations.

Taken together, the present real-time MRI findings unravel a well-orchestrated temporal pattern of physiological swallowing events during deglutition, as summarized in Figure 5. It includes 5 valve function events (VCT, OOT, ERT, GCT, and EOT) and three bolus transport events (OTT, PDT, and PTT), all of which are of particular diagnostic relevance in clinical practice. For example, VCTs happen shortly before OTTs (about 50 ms) and PTTs (about 100 ms), while VCTe happens after OTTe (about 500 ms) and PTTe (about 150 ms). In other words, the VCT covers OTT and PTT in order to fully protect the airway from aspiration by closing the velum and posterior pharyngeal wall. A similar behavior of GCT, namely, a coverage of EOT by closing the glottis and supraglottis could also be detected with GCTs about 80 ms ahead of EOTs and GCTe about 250 to 300 ms later than EOTe. The current durations for GCT (586 ± 93 ms) and PCT (410 ± 73 ms) agree well with previous data from videofluoroscopy, where GCT values have been reported to range between 500 ms [3] and 700 ms [25], and PCT values around 450 ms [26]. The largest deviation from previous studies with values of 400 ms [3] and 500 ms [26] was observed for the EOT duration (261 ± 62 ms). This deviation may be caused by an overestimation of the EOT duration in videofluoroscopy which might be due to a temporally less precise separation of the esophageal entrance from the hypopharyngeal (postcricoid) region.

A major difference between real-time MRI and FEES is the upright body position for FEES and the supine position for MRI. Because statistics based on VCT duration revealed a significant difference between both methods, it seems that the supine position may have some impact on the exact time course of swallowing. This finding needs further investigation of systematic impact of the body position on deglutitive timings. However, any direct comparison will be compromised by the limited number of visible events in FEES and the technical restrictions precluding a simultaneous dual-modality examination. On the other hand, patient studies of swallowing in the supine position have been reported [2, 7], but special care should be taken for patients with neurological disorders to avoid possible complications from aspiration.

In conclusion, the results of this preliminary real-time MRI study at 41 ms temporal resolution offer comprehensive information about the physiology of normal swallowing and the function of the dynamic events. Real-time MRI contributes to our understanding of swallowing by providing images in arbitrary orientations that cover the entire oropharyngolaryngeal region. It also allows for the establishment of new landmarks and standards and provides access to a detailed description of the temporospatial pattern of the swallowing process. The present reference values for normal swallowing in the supine position will serve as the basis for further investigations of pathological conditions as in an ongoing study of dysphagia patients. Other real-time MRI applications may address therapeutic interventions such as functional swallowing therapies. In general, clinical studies are now warranted to assess the real-time MRI potential for diagnosis and treatment monitoring of swallowing disorders.

## Supplementary Material

Supplementary Movie 1: Real-time MRI of normal swallowing at 25 frames per second. Retrospectively synchronized movies of three separate swallows (27-year-old female) in a mid-sagittal, coronal, and axial plane.
Online Supporting Information S7. The related R codes are available.

## Figures and Tables

**Table 1:** Swallowing events revealed by real-time MRI and videofluoroscopy.

Deglutition events	Real-time MRI	Videofluoroscopy [3]
Valve function		
Oro-velar opening (OOT*, reference)	Full rise of soft palate from dorsum	Not defined
Velar-pharyngeal closure (VCT)	Contact of soft palate and pharyngeal wall	Velopharyngeal sphincter (valve 3)
Glottal closure (GCT)	Closure of glottis and supraglottis	Larynx: vocal folds (valve 4c)
Epiglottic retroflexion (ERT)	Contact of epiglottis and supraglottis	Larynx: epiglottis and arytenoid to base of epiglottis (valve 4a/b)
Esophageal opening (EOT)	Separation of postcricoid and pharyngeal wall	Cricopharyngeal sphincter (valve 6)
Bolus transport		
Oro-velar transit (OTT)		Oral transit
	s: OOTs	s: initiation of tongue movement
	e: OOTe	e: bolus head reaches the cross of mandible and tongue base
Pharyngeal delay (PDT)	s: OTTs (OOTs)	s: bolus head reaches cross of mandible and tongue base
	e: PTTs	e: start of laryngeal ascent
Pharyngeal transit (PTT)	s: bolus head passes oro-velar valve	s: start of laryngeal ascent
	e: bolus tail passes esophageal sphincter	e: bolus tail passes cricopharyngeal region
Other		
Laryngeal ascent (LAT)	Ascendance of larynx	Upward and forward movement of hyoid and larynx
Laryngeal descent (LDT)	Descendance of larynx	Not defined
Vallecula and piriform sinus filling (SFT)	Bolus filling vallecula and piriform sinus	Not observed
Pharyngeal constriction (PCT)	Progressive contraction of pharyngeal constrictor	Tongue base and pharyngeal wall (valve 5). Not observed [3]

**Table 2:** Detectability of swallowing events by real-time MRI and FEES.

Deglutition events		Real-time MRI			FEES
		Sagittal	Coronal	Axial	Axial
Laryngeal ascent (LAT)	Start	**100%**	80%	0%	89%
	End	**100%**	67%	0%	
Velo-pharyngeal closure (VCT)	Start	**100%**	***93%***	5%	**100%**
	End	**100%**	*93% *	5%	**100%**
Oro-velar opening (OOT)	Start	**100%**	53%	0%	33%
	End	**100%**	7%	0%	
Pharyngeal transit (PTT)	Start	**100%**	93%	0%	
	End	**100%**	20%	0%	
Glottal closure (GCT)	Start	0%	**87%**	***86%***	
	End	0%	**87%**	***86%***	22%
Vallecular and piriform sinus filling (SFT)	Start	11%	67%	**95%**	
	End	0%	33%	**95%**	
Epiglottic retroflexion (ERT)	Start	**100%**	0%	5%	
	End	**100%**	0%	0%	89%
Pharyngeal constriction (PCT)	Start	**100%**	***80%***	5%	
	End	**100%**	***80%***	5%	
Esophageal opening (EOT)	Start	**95%**	33%	68%	
	End	**95%**	13%	68%	
Laryngeal descent (LDT)	Start	**100%**	47%	0%	
	End	**100%**	53%	0%	11%

Bold font: highest visibility rate.

Bold italic font: second highest visibility rate.

## Abbreviations

EOT: Eesophageal opening
ERT: Epiglottic retroflexion
FEES: Flexible endoscopic evaluations of swallowing
FLASH: Fast low-angle shot
GCT: Glottal closure
LAT: Laryngeal ascent
LDT: Laryngeal descent
MRI: Magnetic resonance imaging
OOT: Oro-velar opening
OTT: Oro-velar transit
PCT: Pharyngeal constriction
PDT: Pharyngeal delay
PTT: Pharyngeal transit
SFT: Vallecular and piriform sinus filling
VCT: Velo-pharyngeal closure.
